# Dilated cardiomyopathy with a MYH6 variant of uncertain significance (c.2894A > C, p.K965 T): a 13-year longitudinal follow-up case report

**DOI:** 10.3389/fcvm.2026.1890762

**Published:** 2026-08-12

**Authors:** Jingwen Deng, Xiaoting Li, Taihao Wang, Xulu Tian, Xiaoyan Cui, Yu Wang, Jiejun Zhang, Rui Chen, Fangqiu Xie, Xuri Zhang, Tao Liu, Yunguang Cen

**Affiliations:** Centre of Geriatrics, Hainan Affiliated Hospital of Hainan Medical University (Hainan General Hospital), Haikou, China

**Keywords:** cardiac magnetic resonance, dilated cardiomyopathy, genotype–phenotype association, late gadolinium enhancement, MYH6, variant of uncertain significance

## Abstract

**Background:**

Genetic evaluation has become a routine component of clinical practice for dilated cardiomyopathy (DCM). However, a substantial proportion of test results return as variants of uncertain significance (VUS), posing considerable challenges for clinical decision-making and genetic counseling. The *MYH6* gene encodes the cardiac *α*-myosin heavy chain, and although its association with DCM has been established, related case reports remain scarce, and the genotype–phenotype correlation is poorly defined.

**Case report:**

We report a 41-year-old Han Chinese male carrying a heterozygous *MYH6* variant (c.2894A > C, p.K965 T), classified as VUS in ClinVar, who presented with a progressive DCM phenotype. Despite 13 years of guideline-directed medical therapy, the patient demonstrated relentless left ventricular enlargement (left ventricular end-diastolic diameter increasing from 71 mm to 98 mm), persistent severe systolic dysfunction (left ventricular ejection fraction consistently ≤17%), and suffered an out-of-hospital cardiac arrest in the 12th year of his disease course. Cardiac magnetic resonance (CMR) revealed extensive transmural septal fibrosis. Sanger sequencing of family members showed that his healthy mother carried the identical variant, whereas his affected father and elder brother did not, forming a classic “genotype–phenotype non-segregation” paradox. Following the cardiac arrest, the patient received a cardiac resynchronization therapy defibrillator (CRT-D), but exhibited no left ventricular reverse remodeling at 12 months post-implantation and is currently undergoing pre-transplant evaluation for heart transplantation.

**Conclusion:**

This case suggests that a *MYH6* VUS may be associated with a severe and rapidly progressive DCM phenotype in specific clinical contexts. However, the non-segregation within the family implies that this variant may act merely as a disease modifier or may require synergistic genetic or environmental co-factors to manifest pathogenicity. Long-term multimodality imaging follow-up and assessment of device therapy response are critical for individualized management of VUS carriers.

## Introduction

Dilated cardiomyopathy (DCM) remains one of the leading causes of heart failure and sudden cardiac death, with an estimated 30%–50% of cases having a genetic basis (1, 2). With the widespread adoption of next-generation sequencing, genetic testing has become an integral component of the etiological workup for DCM. However, an increasingly prominent clinical dilemma is that a considerable proportion of test results are not classified as clearly pathogenic or benign, but rather as variants of uncertain significance (VUS). It is estimated that VUS are identified in 15%–30% of genetic tests for cardiomyopathies, generating substantial interpretive ambiguity and management uncertainty for clinicians, patients, and their families alike (3).

The *MYH6* gene encodes the cardiac *α*-myosin heavy chain, a core component of the sarcomeric thick filament. Pathogenic variants in this gene were initially linked to hypertrophic cardiomyopathy and atrial septal defects, and their association with DCM was first established by Carniel et al. in 2005, with a detection rate of approximately 4% in DCM cohorts (4, 5). Most reported *MYH6*-related DCM cases exhibit a late-onset, slowly progressive clinical course. Nevertheless, the phenotypic heterogeneity of *MYH6*-associated cardiomyopathies is substantial, and a large number of reported variants remain classified as VUS, warranting further case accumulation and functional validation (6).

Herein, we present 13-year longitudinal follow-up data from a patient with progressive DCM carrying the *MYH6* c.2894A > C (p.K965T) VUS. The distinctive value of this case lies in its demonstration of three paradoxes: (1) a progressive clinical phenotype that deviates from the previously described indolent course of *MYH6*-related DCM; (2) a severe phenotype contrasting with non-segregation within the family, as the healthy mother carries the same variant; and (3) extensive myocardial fibrosis coupled with non-response to CRT-D therapy. Through this report, we aim to explore how the integration of serial imaging, device therapy response, and pedigree data can inform individualized clinical management in the context of a VUS.

## Case report

### Initial presentation and baseline assessment

A 41-year-old Han Chinese male presented to our institution in March 2013 with a 2-week history of exertional chest tightness and dyspnea. He had a long-standing smoking history (1 pack/day) and occasional alcohol consumption, with no history of hypertension, diabetes mellitus, connective tissue disease, or recent viral infection. His mother and elder brother were reportedly healthy on routine check-ups, with no known family history of cardiomyopathy or sudden cardiac death. His father was later found to be affected by cardiomyopathy.

**Figure 1:**
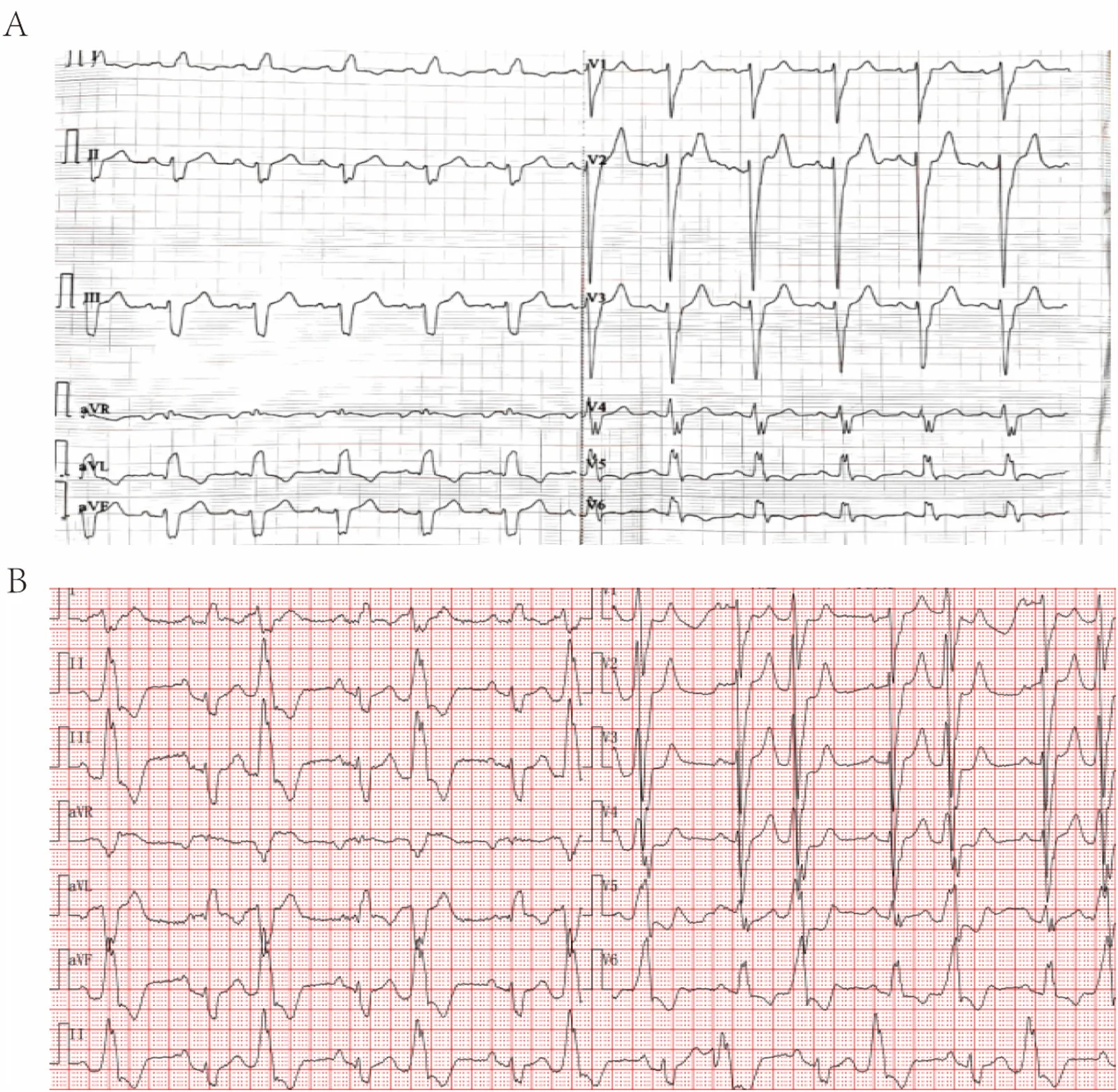
**(A)** the patient's ECG in 2013 revealed sinus rhythm with CLBBB. **(B)** ECG of the patient after cardiac arrest in 2025 showing sinus rhythm, CLBBB, QRS duration 178 ms, and frequent ventricular premature contractions.

On admission, physical examination revealed a heart rate of 120 beats/min, mild jugular venous distension, and bilateral basal crackles on pulmonary auscultation. Electrocardiography (ECG) demonstrated complete left bundle branch block (CLBBB) (Figure 1A). Transthoracic echocardiography (TTE) showed severe left ventricular enlargement [left ventricular end-diastolic diameter (LVEDD) 71 mm] and a left ventricular ejection fraction (LVEF) of 14%. Coronary angiography excluded obstructive coronary artery disease. Laboratory testing revealed an N-terminal pro-brain natriuretic peptide (NT-proBNP) level of 3,339 ng/L (reference <125 ng/L). Thyroid function tests, anti-streptolysin O titer, interferon-gamma release assay, anti-double-stranded DNA antibodies, D-dimer, HIV serology, and viral panels for Coxsackievirus, adenovirus, cytomegalovirus, and Epstein–Barr virus were all negative.

A diagnosis of DCM was established, and guideline-directed medical therapy (GDMT) was initiated: benazepril 10 mg qd, spironolactone 20 mg qd, and bisoprolol starting at 1.25 mg qd. At discharge, the patient's symptoms had improved, with functional recovery to New York Heart Association (NYHA) class II.

### Disease progression and therapeutic adjustments

By May 2018, the patient's cardiac function had progressively declined to NYHA class III. Follow-up TTE revealed further left ventricular enlargement (LVEDD 86 mm), persistently severely depressed LVEF (12%), and an NT-proBNP elevation to 4,520 ng/L. According to contemporary guidelines, the patient met class I indications for primary prevention with a cardiac resynchronization therapy defibrillator (CRT-D): sinus rhythm, persistent CLBBB, QRS duration ≥150 ms, LVEF ≤35% despite at least 3 months of optimized medical therapy, and NYHA class III. The multidisciplinary heart failure team thoroughly explained the potential benefits and risks of device therapy; however, the patient declined implantation due to personal concerns and financial constraints, opting instead for intensified medical therapy. The regimen was modified as follows: benazepril was switched to sacubitril/valsartan 100 mg bid; bisoprolol was up-titrated to the target dose of 10 mg qd (tolerated without significant adverse effects); dapagliflozin 10 mg qd was added; and furosemide 20 mg was prescribed on an intermittent basis. Following these adjustments, the patient experienced partial symptomatic improvement (NYHA class II) and NT-proBNP transiently decreased to 2,036 ng/L, but LVEF remained at only 13%.

### Advanced heart failure and genetic evaluation

In July 2023, 10 years after the initial presentation, the patient was readmitted with recurrent heart failure manifesting as orthopnea, peripheral edema, and NYHA class IV symptoms, complicated by cardiogenic shock (blood pressure 80/60 mmHg). Laboratory studies revealed renal impairment (creatinine 180 μmol/L) and markedly elevated NT-proBNP (>22,388 ng/L). TTE demonstrated further enlargement of LVEDD to 94 mm with an LVEF of 12%. The patient was admitted to the cardiac intensive care unit and received levosimendan for inotropic support and high-dose diuretic therapy. After stabilization, oral torasemide 10 mg qd and vericiguat 2.5 mg qd were initiated. Given the patient's recurrent NYHA class IV refractory heart failure, LVEDD >90 mm, LVEF <20%, recurrent cardiogenic shock, and malignant ventricular arrhythmias, the multidisciplinary team strongly recommended referral to an advanced heart failure center for evaluation of left ventricular assist device (LVAD) or heart transplantation, in accordance with the 2023 ESC cardiomyopathy guidelines (7). However, the patient again declined on personal and financial grounds.

**Figure 2:**
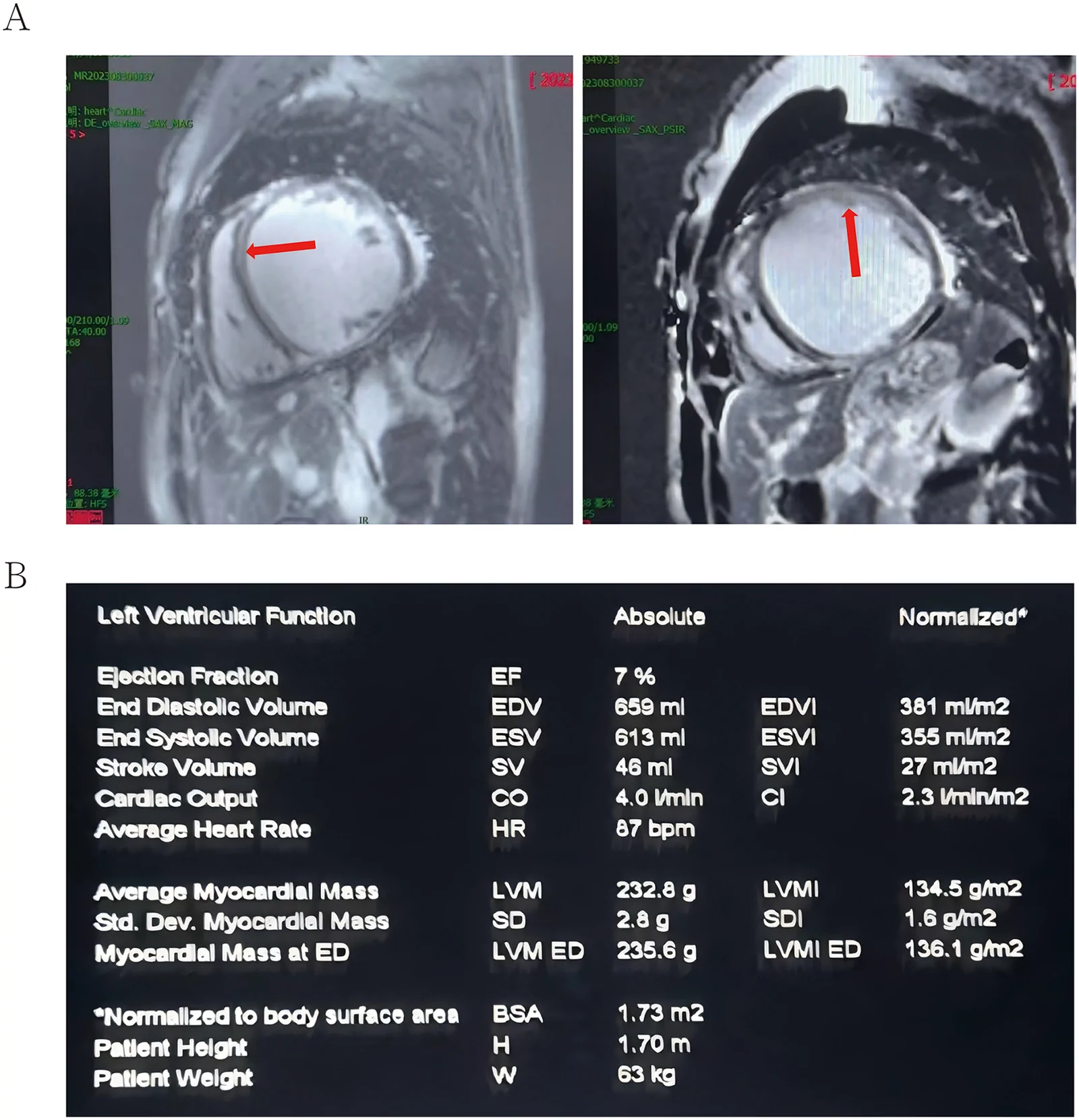
Cardiac magnetic resonance (CMR) imaging performed in 2023. **(A)** Short-axis steady-state free precession (SSFP) cine image demonstrates severe biventricular enlargement with marked thinning of the left ventricular wall. Late gadolinium enhancement (LGE) sequence reveals extensive transmural hyperenhancement involving the interventricular septum (red arrows), along with scattered spotty and subendocardial linear hyperenhancement in the left ventricular free wall. **(B)** Quantitative CMR left ventricular functional analysis report. The data show a severely depressed ejection fraction of 7%, indicating profound myocardial pump failure; an end-diastolic volume index (EDVI) of 381 mL/m^2^, reflecting marked spherical left ventricular dilatation; and increased myocardial mass, consistent with advanced adverse remodeling in end-stage dilated cardiomyopathy.

In August 2023, cardiac magnetic resonance (CMR) was performed, revealing severe cardiac enlargement (left ventricular maximum transverse diameter approximately 83 mm and maximum longitudinal diameter 117 mm; right ventricular maximum transverse diameter approximately 35 mm and maximum longitudinal diameter 69 mm), with a left ventricular end-diastolic volume of 659 mL (end-diastolic volume index 381 mL/m²) and an LVEF of 7%. First-pass perfusion imaging showed scattered patchy hypoperfusion areas involving the entire left ventricular circumference. Late gadolinium enhancement (LGE) demonstrated scattered spotty and linear hyperenhancement involving the subendocardial and intramural layers of the interventricular septum and the left ventricular free wall, with most regions exhibiting transmural enhancement (Figure 2).

**Figure 3:**
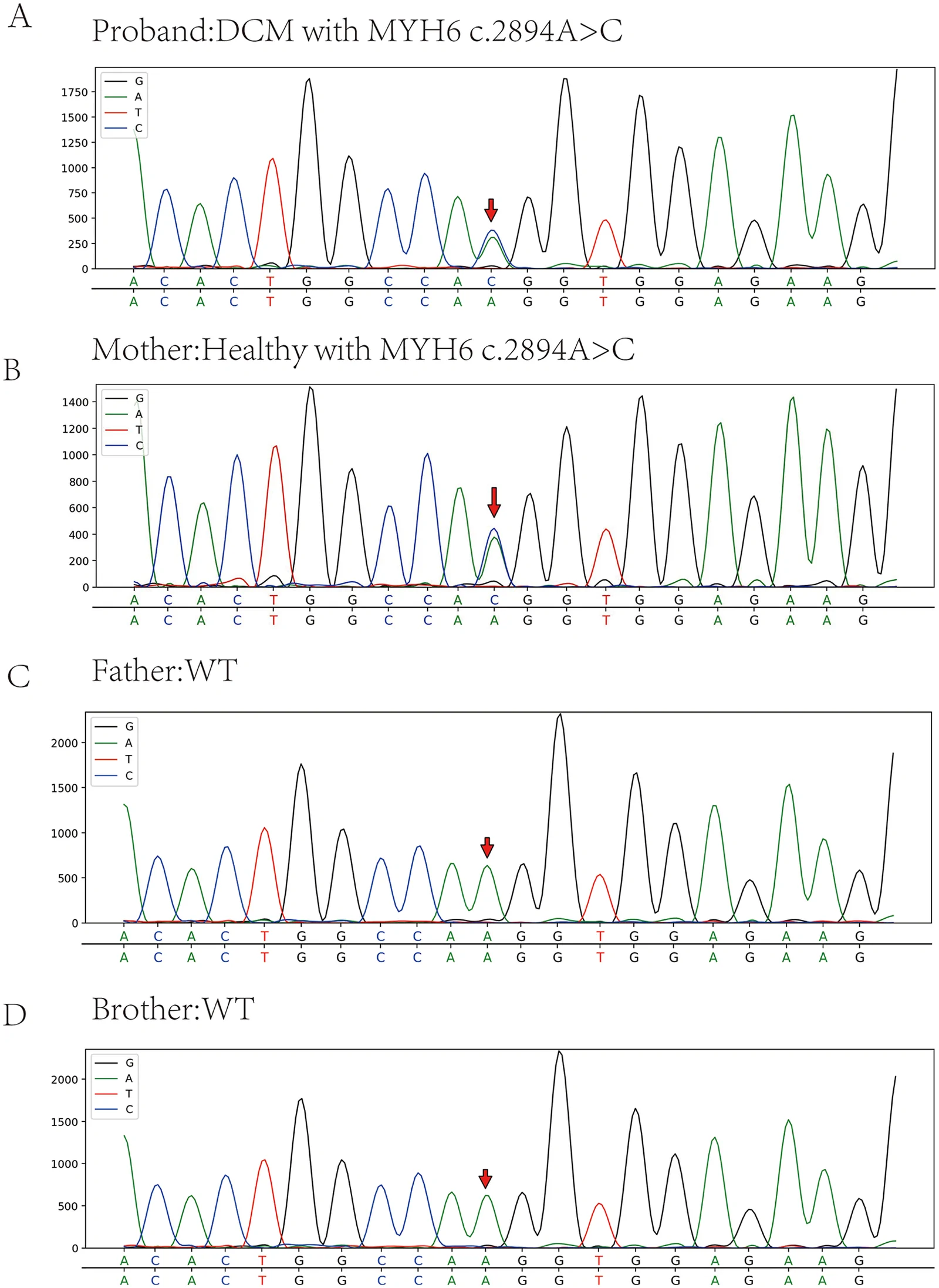
Sanger Sequencing validation of the *MYH6* variant. Sanger sequencing chromatograms demonstrate a heterozygous c.2894A > C (p.K965T) variant in the proband and his phenotypically healthy mother **(A,B)**, while the affected father and the unaffected elder brother **(C,D)** both carry the wild-type allele.

In September 2024, clinical whole-exome sequencing (WES) was performed, covering all protein-coding exonic regions of the human genome. A heterozygous missense variant, c.2894A > C (p.K965T), was identified in exon 22 of the *MYH6* gene (NM_002471.4). This variant is not documented in the HGMD database and is classified as VUS in ClinVar. No additional pathogenic or likely pathogenic variants related to the phenotype were detected. Sanger sequencing of family members revealed that the patient's healthy mother carried the same variant, whereas his affected father and unaffected younger brother did not (primer sequence: CAGGAAACAGCTATGACCATGCCAAGGACCAGGACAACAC), establishing a clear “genotype–phenotype non-segregation” pattern (Figure 3).

### Device implantation and follow-up

**Figure 4:**
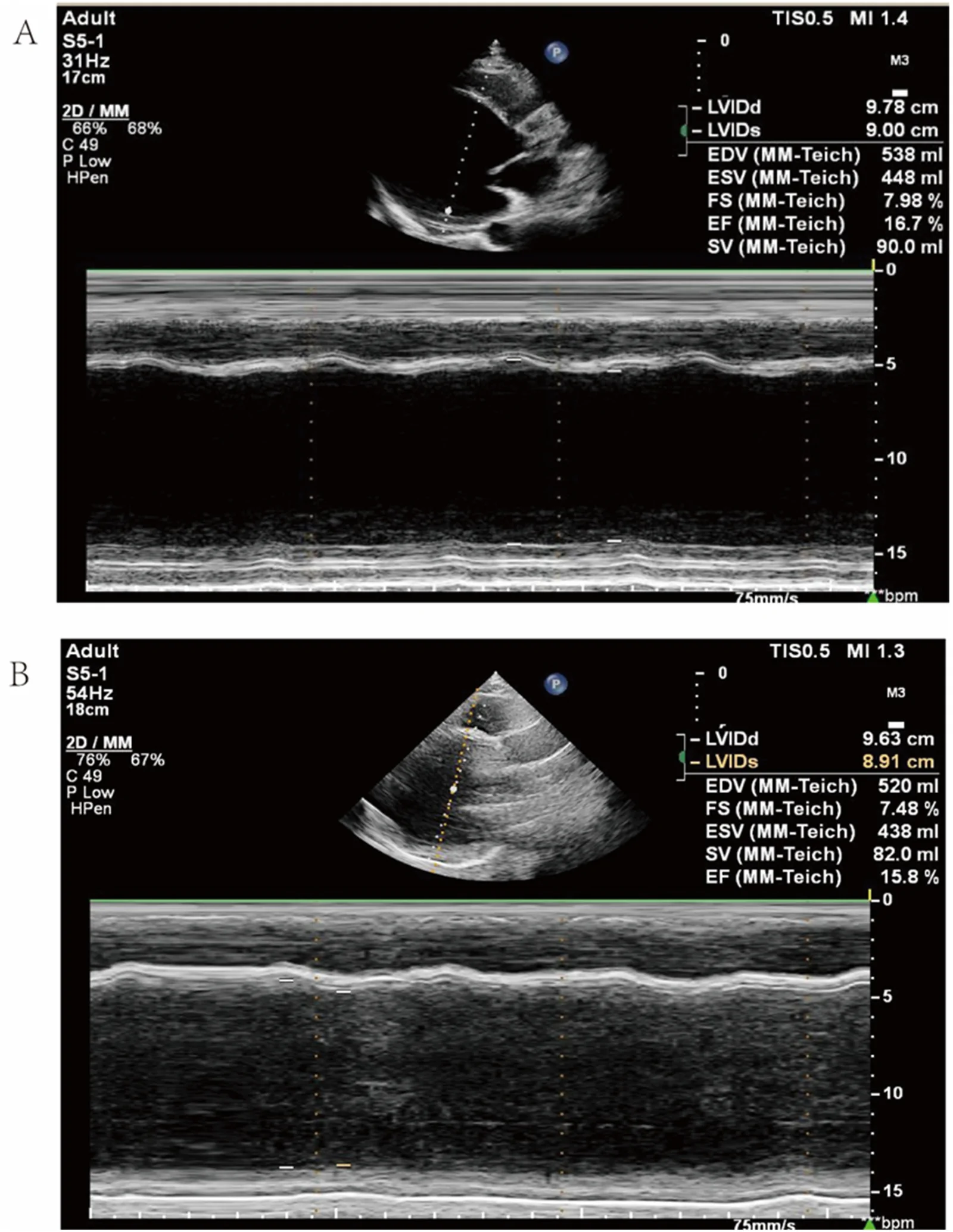
**(A)** TTE of the patient in 2025 showing severe left ventricular enlargement (LVEDD 98 mm), severe systolic dysfunction, and LVEF 17%). **(B)** TTE of the patient in 2026 showing severe left ventricular enlargement (LVEDD 96 mm), severe systolic dysfunction, and LVEF 16%.

In February 2025, the patient experienced an out-of-hospital cardiac arrest and was successfully resuscitated. Post-resuscitation ECG still showed CLBBB with a QRS duration of 178 ms and frequent premature ventricular contractions (Figure 1B). TTE demonstrated an LVEDD of 98 mm and LVEF of 17% (Figure 4A). Based on the 2023 ESC guideline criteria (LVEF ≤35%, NYHA class II–IV, LBBB morphology with QRS ≥150 ms), the patient met class I indications for CRT-D as secondary prevention. Consequently, a CRT-D device was successfully implanted on March 3, 2025, after which his symptoms improved to NYHA class II–III.

During the 12-month follow-up period post-CRT-D implantation, the patient self-reported three shock episodes (detailed data were unavailable due to objective reasons). Despite long-term tolerance of maximized GDMT (sacubitril/valsartan 200 mg bid, bisoprolol 10 mg qd, torasemide 20 mg bid, dapagliflozin 10 mg qd, vericiguat 10 mg qd, and finerenone 10 mg qd), the patient experienced an episode of heart failure exacerbation requiring hospitalization for intravenous levosimendan after self-reducing the torasemide dose due to perceived low blood pressure. The frequency of heart failure hospitalizations continued to rise, with a total of 4 admissions within 1 year. Follow-up echocardiography in 2026 showed persistent severe left ventricular enlargement (LVEDD 96 mm) with an LVEF of 16% (Figure 4B), indicating no significant reverse remodeling compared with pre-implantation status. The patient is currently undergoing pre-heart transplantation evaluation.

## Discussion

This case report delineates the 13-year clinical trajectory of a patient with DCM harboring the *MYH6* c.2894A > C (p.K965T) VUS. The constellation of a rapidly progressive phenotype, intra-familial genetic non-segregation, and non-responsiveness to cardiac resynchronization therapy provides a unique window into the multifaceted complexity of *MYH6*-related cardiomyopathies and underscores the considerable challenges that VUS poses in contemporary cardiovascular genetics.

### The MYH6 gene and dilated cardiomyopathy: current evidence

*MYH6* encodes the *α*-myosin heavy chain, a major component of the cardiac sarcomere. While pathogenic variants in *MYH6* are well-established causes of hypertrophic cardiomyopathy and congenital heart defects (particularly atrial septal defects), their role in DCM is less firmly established. Carniel et al. first reported *MYH6* mutations in DCM cohorts with an estimated prevalence of ∼4% (4). Subsequent studies have identified various *MYH6* variants in DCM patients, but the gene–disease validity for autosomal dominant DCM remains at a “moderate” or “limited” level in some gene curation frameworks. Most reported *MYH6*-related DCM cases exhibit a late-onset, slowly progressive clinical course, although phenotypic heterogeneity is substantial (5). Importantly, many reported variants remain classified as VUS, underscoring the need for functional studies and larger cohort analyses to definitively establish causality (6).

### The VUS dilemma and family non-segregation

The most compelling genetic finding in this family is the complete non-segregation of the variant with the disease phenotype: the patient's healthy mother carries the same heterozygous variant, while his affected father and unaffected brother do not. This pattern effectively rules out a simple Mendelian autosomal dominant inheritance model with complete penetrance. Importantly, the absence of the variant in the affected father and its presence in the healthy mother strongly argue against this VUS being the primary monogenic cause of this family's DCM.

This observation does not entirely exclude the possibility that the variant contributes to disease as a modifier or in combination with other genetic or environmental factors (8). However, it delivers a sobering reminder that familial segregation data must be interpreted with extreme caution in the context of VUS, and that a single pedigree should never be used as the sole basis for pathogenicity classification. The case underscores the value of thoroughly phenotyping all available family members—including obligate carriers—to refine variant interpretation and avoid overattributing clinical phenotypes to VUS.

### The prognostic value of cardiac magnetic resonance

From an imaging perspective, the extensive transmural septal fibrosis documented by CMR constitutes a pivotal finding with direct therapeutic and prognostic implications. The presence and extent of late gadolinium enhancement have been repeatedly validated as powerful independent predictors of adverse outcomes in DCM, particularly for sudden cardiac death and ventricular tachyarrhythmias (9, 10). In our patient, the transmural distribution of fibrosis—spanning the full thickness of the interventricular septum—offers a compelling explanation for his poor response to CRT-D. Extensive scar tissue not only impedes the mechanical efficacy of biventricular pacing by replacing viable myocardium with inexcitable fibrous tissue, but also serves as an arrhythmogenic substrate that perpetuates reentrant circuits, a phenomenon concordant with the patient's recurrent appropriate shocks and the complete absence of left ventricular reverse remodeling at 12 months post-implantation.

It is crucial to distinguish between the prognostic implications of LGE and the established indications for CRT-D. While LGE burden provides important prognostic information regarding arrhythmic risk and disease progression, the decision to implant a CRT-D should be based on established clinical criteria—LVEF, NYHA functional class, QRS duration and morphology—rather than on the presence or pattern of LGE alone (7). In this case, the patient met class I criteria for CRT-D on multiple occasions based on these clinical parameters, independent of his genetic findings. The extensive LGE may, however, help explain his suboptimal response to CRT-D and underscores the importance of pre-implant CMR in setting realistic expectations for reverse remodeling.

### Clinical management implications and therapeutic timing

The therapeutic odyssey of this patient also illuminates the profound management dilemmas engendered by a VUS result. Despite meeting well-established guideline criteria for device therapy on multiple occasions, the patient deferred or declined CRT-D implantation, partly motivated by uncertainty regarding the anticipated benefit—a hesitation that is directly entangled with the ambiguous clinical significance of his genetic finding. When genetic testing fails to provide a clear pathogenic or prognostic classification, patients and clinicians alike are left navigating a grey zone in which decision-making must pivot away from genotype-centric algorithms and toward dynamically re-assessed phenotypic variables.

Critically, the patient met guideline indications for CRT-D as primary prevention as early as 2018, based on LVEF ≤35%, NYHA class III, and CLBBB with QRS duration ≥150 ms. The delayed implantation until after cardiac arrest in 2025 represents a missed opportunity for primary prevention, independent of any genetic consideration. This underscores that device therapy decisions should be driven by established clinical risk stratification tools, not by the presence or absence of a particular genetic variant.

### Limitations

Several limitations merit acknowledgment. First, as a single case observation, our findings are inherently hypothesis-generating and not generalizable to the broader DCM population. Second, the absence of *in vitro* functional assays or animal models precludes definitive mechanistic insights into the molecular effect of the c.2894A > C (p.K965T) variant. Third, incomplete retrieval of device interrogation data limited our ability to precisely characterize the burden and triggers of ventricular arrhythmias post-CRT-D implantation. Fourth, the patient's repeated refusals of invasive procedures prevented acquisition of endomyocardial biopsy or hemodynamic data that could have further contextualized the disease mechanism. Fifth, the patient's long-standing smoking history and occasional alcohol consumption represent confounding environmental factors that cannot be excluded as contributors to disease progression. Finally, a comprehensive genetic evaluation of additional DCM-related genes (such as *LMNA*, *DES*, *FLNC*, and *TTN*) in the patient's family members was not performed, which limits our ability to assess for potential oligogenic inheritance. Future collaborative studies incorporating larger DCM cohorts, comprehensive genomic analyses, and systematic functional validation will be essential to clarify the clinical relevance of *MYH6* VUS and to refine the classification of such variants in clinical practice.

## Conclusion

This case report demonstrates a triple clinical dilemma in a DCM patient carrying the *MYH6* VUS (c.2894A > C, p.K965T) over 13 years of follow-up: rapidly progressive phenotype, familial non-segregation, and non-response to CRT-D. This case emphasizes that VUS interpretation should integrate longitudinal phenotypic data, pedigree information, and advanced imaging biomarkers, rather than relying solely on the classification provided in genetic testing reports. The presence of extensive transmural LGE on CMR provides valuable prognostic information and may help explain poor response to CRT-D. For such patients, individualized multidisciplinary management and timely referral to an advanced heart failure center—based on clinical criteria rather than genetic classification alone—are paramount. Future research should focus on functional validation of this variant and assessment of the clinical relevance of *MYH6* VUS in larger DCM cohorts.

## Data Availability

The datasets presented in this study can be found in online repositories. The names of the repository/repositories and accession number(s) can be found in the article/Supplementary Material.
